# Antimicrobial and Antibiofilm Potential of Green-Synthesized Graphene–Silver Nanocomposite against Multidrug-Resistant Nosocomial Pathogens

**DOI:** 10.3390/biomedicines12051104

**Published:** 2024-05-16

**Authors:** Preeti Negi, Jatin Chadha, Kusum Harjai, Vijay Singh Gondil, Seema Kumari, Khem Raj

**Affiliations:** 1 Department of Microbiology, Basic Medical Sciences Block 1, South Campus, Panjab University, Sector-25, Chandigarh 160014, India; 2 Department of Microbiology & Immunology, School of Medicine and Dentistry, University of Rochester, Rochester, NY 14642, USA

**Keywords:** multidrug-resistant pathogens, hospital-acquired infections, graphene oxide, green synthesis of nanoparticles, antibacterial and antifungal, *Candida* spp., *Pseudomonas aeruginosa*

## Abstract

Hospital-acquired infections (HAIs) pose a significant risk to global health, impacting millions of individuals globally. These infections have increased rates of morbidity and mortality due to the prevalence of widespread antimicrobial resistance (AMR). Graphene-based nanoparticles (GBNs) are known to possess extensive antimicrobial properties by inflicting damage to the cell membrane, suppressing virulence, and inhibiting microbial biofilms. Developing alternative therapies for HAIs and addressing AMR can be made easier and more affordable by combining nanoparticles with medicinal plants harboring antimicrobial properties. Hence, this study was undertaken to develop a novel graphene–silver nanocomposite via green synthesis using *Trillium govanianum* plant extract as a reducing agent. The resulting nanocomposite comprised silver nanoparticles embedded in graphene sheets. The antibacterial and antifungal properties of graphene–silver nanocomposites were investigated against several nosocomial pathogens, namely, *Candida auris*, *Candida glabrata*, *Escherichia coli*, *Staphylococcus aureus*, *Klebsiella pneumoniae*, and *Pseudomonas aeruginosa*. The nanocomposite displayed broad-range antimicrobial potential against the test pathogens, with minimum inhibitory concentrations (MICs) ranging between 31.25 and 125.0 µg/mL, and biofilm inhibition up to 80–96%. Moreover, nanocomposite-functionalized urinary catheters demonstrated hemocompatibility towards sheep erythrocytes and imparted anti-fouling activity to the biomaterial, while also displaying biocompatibility towards HEK 293 cells. Collectively, this investigation highlights the possible application of green-synthesized GBNs as an effective alternative to conventional antibiotics for combating multidrug-resistant pathogens.

## 1. Introduction

Hospital-acquired infections (HAIs), particularly those caused by drug-resistant pathogens, pose a significant global health threat. They affect more than 100 million people worldwide and 4 million patients in developed countries each year, with significant consequences for patient safety and disease burden [1]. HAIs are associated with higher rates of morbidity and mortality, longer hospital stays, and significant financial costs. According to the World Health Organization (WHO) estimates, these infections account for 4–56% of the neonatal deaths, with high incidence rates in Southeast Asia and Sub-Saharan Africa [2]. In developed countries, the prevalence reaches 37% among patients admitted to intensive care units [1]. HAIs often involve multidrug-resistant bacterial pathogens such as *Streptococcus* spp., *Acinetobacter* spp., *Enterococci*, *Pseudomonas aeruginosa*, coagulase-negative *Staphylococci* (CoNS), *Staphylococcus aureus*, *Bacillus cereus*, *Legionella*, and other members of the *Enterobacteriaceae* family [1]. Among opportunistic infections caused by fungi, *Candida* spp. is the most prevalent, followed by *Aspergillus* spp., in intermediate to severely immunocompromised individuals, and less common molds, such as *Mucor* spp. and *Fusarium* spp., in severely immunocompromised patients [3]. Biofilm formation contributes to increased pathogenicity through reduced penetration of antimicrobial drugs, thereby playing a major role in persistent outbreaks of HAIs. Global estimates indicate that over 1.2 million deaths in 2019 were directly related to antimicrobial resistance (AMR) [4]. If insufficient action is taken to control AMR, this number is expected to rise to about 10 million deaths annually by 2050 [5]. Moreover, the coronavirus disease 2019 (COVID-19) pandemic worsened the current scenario of AMR due to the unimaginable rise in hospital admissions and heavy prescription of antimicrobial therapy in diseased individuals [6,7,8]. However, it has been insinuated that such antimicrobial treatment may not always be appropriate, as it could lead to an increase in drug resistance worldwide [8]. Hence, AMR, also known as the ‘silent pandemic’, poses a much larger problem to mankind, demanding immediate attention, coordinated management, and vigilance among the healthcare sectors [5].

The investigation of metallic nanoparticles, particularly against multidrug-resistant microbes, has increased exponentially in recent years. Although there are a variety of chemical methods used for formulating nanoparticles, ‘green synthesis/approaches’ have gained considerable interest due to their environmental benefits, low energy consumption, and potential to address the menace of drug resistance [9]. In this context, combining nanoparticles with medicinal plants exhibiting antimicrobial properties is a cost-effective and simple technique. Plant extracts contribute to metal ion reduction, particle-shape formation, and colloidal stability, consequently augmenting their antifungal and antibacterial properties [10,11]. *Trillium govanianum*, a trans-Himalayan medicinal plant with recently described antimicrobial potential [12], has been used as a reducing agent for green synthesis of silver-based nanoparticles [13]. Govanoside A, Borassoside E, pennogenin, and diosgenin have been reported to exhibit good antifungal activity against *Aspergillus niger*, *Candida albicans*, and *Candida glabrata* [14]. The antibacterial, antiviral, and antioxidant qualities of *T. govanianum* have been attributed to the abundance of phytoconstituents, such as saponins and alkaloids [15]. Further, nanoparticles extend broad-range antimicrobial prospects through various mechanisms, including membrane damage, suppression of virulence factors, inhibition of microbial enzymes and toxins, and abolishment of biofilm formation. Due to their larger size and more robust defense and repair systems than microorganisms, human cells are generally less susceptible to nanoparticles [16]. However, cytotoxic effects have also been documented, particularly when nanoparticles are employed at higher concentrations [17]. Toxicity with silver nanoparticles (AgNPs) is extensively reported in in vitro studies, but these effects vary drastically in complex organisms [18]. Nevertheless, the practical application of AgNPs becomes limited due to the loss of antibacterial activity and self-aggregation or precipitation over time [19,20]. Consequently, creating stable dispersed AgNP substrates and managing Ag⁺ release have emerged as crucial challenges in drug delivery. Graphene and its derivatives have demonstrated to be highly suitable options for tackling these issues. According to Li et al., graphene enables the gradual release of silver nanoparticles, thereby preventing self-aggregation or precipitation and overcoming unfavorable off-target effects or cellular toxicity caused by silver accumulation [20].

Graphene-based nanoparticles (GBNs) have recently gained attention as drug-delivery vehicles due to their unique properties, including a large surface-to-volume ratio, mechanical flexibility, and thermal stability [21]. Graphene, a 2D carbon-based material with a honeycomb appearance, induces mechanical stress and prevents microbial cell development. Graphene oxide nanocomposites have been used for local antifungal activity, wound regeneration, and the targeting of specific infections in mucocutaneous tissues [22]. These have also been functionalized with antimicrobial peptides for imparting broad-spectrum antimicrobial and anti-fouling properties against nosocomial bacterial pathogens [23]. The effects of graphene oxide–silver nanoparticles have been investigated against Gram-positive and Gram-negative bacteria [24], as well as *C. albicans* [20]. These nanoparticles have shown potential in various applications, with minimal toxicity against human cells. Additionally, the biocompatibility of GO towards human cells depends on concentration and sheet morphology, with higher concentrations leading to plasma membrane penetration and increased ROS synthesis [17]. Considering the emerging application of graphene in developing nanoformulations with improved drug delivery, this study explored the potential use of traditional medicine in combination with nanotechnology to develop an alternative therapy to antibacterial and antifungal drugs. Graphene–silver nanocomposites were prepared using a green approach, employing *T. govanianum* plant extract, followed by biophysical characterization to assess their morphological properties and evaluate their antimicrobial potential. The nanoformulation-impregnated silicone urinary catheters were subsequently examined for their hemocompatible and biocompatible nature, followed by evaluating their biological properties in terms of anti-fouling potential against multidrug-resistant microorganisms.

## 2. Materials and Methods

### 2.1. Microorganisms and Growth Media

Clinical isolates of *C. auris* (NCCPF 470197, NCCPF 470200, and NCCPF 470203) and *C. glabrata* clinical (NCCPF 100033, NCCPF 100037) and standard strains (ATCC 2001) were procured from the National Culture Collection of Pathogenic Fungi (NCCPF), Department of Medical Microbiology, Postgraduate Institute of Medical Education and Research (PGIMER), Chandigarh, India. Laboratory-maintained strains of *Escherichia coli*, *Staphylococcus aureus*, *Klebsiella pneumoniae*, and *Pseudomonas aeruginosa* were used in this study. Yeast and bacterial cultures were grown on Sabouraud Dextrose Agar and nutrient agar plates at 30 °C and 37 °C, respectively. The isolates were preserved in 50% glycerol at −80 °C.

### 2.2. Cell Surface Hydrophobicity Assay

Cell surface hydrophobicity (CSH) was assessed using the microbial adhesion assay for hydrocarbons (MATH) [25]. Xylene was used as the solvent to demonstrate the hydrophobic surface characteristic. Cells were grown, harvested, and washed twice with PBS. Cell suspension, displaying an OD between 0.4 and 0.5 at 600 nm, was prepared in 0.1 M KNO_3_ (A_0_); 3 mL of this suspension was overlaid by 1 mL xylene and incubated at 30 °C for 10 min. After vigorous vortexing, phases were allowed to separate for 20 min and the OD of the aqueous phase at 600 nm was measured (A_1_). The percentage of hydrophobicity was calculated as follows:

Hydrophobicity (%) = [1 − (A_1_/A_0_)] × 100

### 2.3. Preparation of Plant Extracts

Fresh rhizomes of *Trillium govanianum* were washed with hydrogen peroxide (3%), followed by double deionized water (ddH_2_O), dried, and surface sterilized before being crushed into a fine powder. Plant extracts were prepared by the Soxhlet extraction method using ethanol as a solvent [26]. The extract was then lyophilized at −50 °C, extraction yield was calculated, and the extract was refrigerated until further use.

### 2.4. Green Synthesis and Characterization of Graphene–Ag Nanocomposites (GO-AgNPs)

#### 2.4.1. Preparation of GO

Graphene oxide (GO) was prepared from powder graphite adopting the Hummer and Offeman method [27]. The product was filtered and washed with acetone to make it moisture-free and dried in an air oven at 65 °C overnight. The GO suspension in water (1 mg/mL) was exfoliated through ultrasonication for 1 h. A homogeneous solution of GO nanosheets was obtained. This GO–water suspension was used as a matrix for the preparation of the Ag nanoparticles.

#### 2.4.2. Preparation of AgNPs on GO

Graphene–silver nanocomposites were synthesized by reducing silver nitrate (AgNO_3_) using a plant extract in the presence of a GO suspension. In a flask, 50 mL of GO suspension was mixed with 100 mL of aqueous AgNO_3_ solution (1 mM). The resulting mixture was stirred for 30 min at room temperature. Subsequently, 100 mL of the plant extract (1 mg/mL) was slowly added to the reaction mixture of AgNO_3_–GO suspension under vigorous stirring. The reaction mixture was sonicated for 30 min to ensure complete reduction [28]. The color of the solution changed as the reaction progressed and was further examined using a UV–vis. spectrophotometer. As the reaction progressed, the color of the solution changed from dark grey to brown-black. The solution was further analyzed using a UV–vis. spectrophotometer.

#### 2.4.3. Characterization of GO-Ag NPs

To detect and confirm the formation of GO-AgNPs during its synthesis via *T. govanianum* extract, UV–visible spectroscopy was performed at room temperature. Fourier transform infrared spectroscopy (FTIR) was used to confirm the capping of the extract on the surface of GO-AgNPs by a Frontier PerkinElmer spectrometer (PerkinElmer, Waltham, MA, USA). The microscopic images of the pristine GO and GO-AgNP were observed using Transmission electron microscopy (TEM) to investigate the morphology of the synthesized nanocomposites. 

### 2.5. In Vitro Antimicrobial Study of Graphene–Ag-Nanocomposite

#### 2.5.1. Agar-Well Diffusion Assay

The antibacterial and antifungal assays were performed using the agar-well diffusion technique [29]. Standard antibiotics (amikacin, gentamicin, and vancomycin) and an antifungal drug (posaconazole) served as positive quality controls, while distilled water was used as a negative control.

#### 2.5.2. Microbroth Dilution Assay

The inhibitory effects of GO-AgNPs on bacterial and fungal isolates were tested on the surface of 96-well microplates according to the Clinical and Laboratory Standards Institute *M27-A2* (CLSI) protocol [30]. Briefly, isolates from the overnight-grown cultures were harvested by centrifugation and adjusted to a final concentration of 1 × 10^6^ cells/mL for *Candida* isolates and 1 × 10^8^ cells/mL for bacterial isolates in PBS. A total of 100 mg/mL GO-Ag NPs solution was added into a 100 µL sterile broth in a 96-well plate and subjected to two-fold serial dilution. Then, 100 µL of test culture was added to each well. After overnight incubation at 37 °C, optical density measurements were taken at 600 nm. The wells treated with the respective antifungal/antibiotic drugs were taken as positive controls while the wells containing only medium served as blank controls. All assays were performed in quadruplets and repeated at least twice.

#### 2.5.3. Quantitative Biofilm Inhibition Assays

The activity of GO-AgNPs on candidal [31] and bacterial [32] biofilm formation was assessed using previously described protocols. Briefly, microbial cultures collected from overnight growth were centrifuged, and the resulting pellets were washed twice with sterile PBS and resuspended in RPMI-1640 (*Candida* spp.) and nutrient broth (bacteria) to attain cell densities of 10^8^ CFU/mL. The diluted cultures were then added to wells of microtiter plates containing serial dilutions of GO-AgNPs (at MIC-MIC/64). The plates were then incubated at 37 °C for 24 h, carefully washed twice with PBS, and the extent of biofilm formation was estimated following staining with crystal violet (CV).

#### 2.5.4. Functionalization of the Catheters with GO-AgNPs

Silicone urinary catheters were modified using a solvent-swelling-based impregnation method [33]. Concisely, the catheter segments of 1 cm each were carefully cut, disinfected with 70% alcohol and sterilized by autoclaving. After being pretreated with chloroform for 2 h at 37 °C, the sterile catheter segments were transferred to a sterile 24-well plate. To every well, 2 mL of GO-AgNP solution was added for functionalizing the catheter surface via impregnation, followed by overnight incubation at 37 °C. Post-incubation, the impregnated catheter segments were removed and gently washed twice using sterile PBS to remove any loosely bound GO-AgNPs.

#### 2.5.5. Field Emission Scanning Electron Microscopy of Microbial Biofilms on GO-AgNP-Functionalized Catheters

The ability of GO-AgNP-impregnated urinary catheters to avert cellular adhesion and biofilm formation by *C. auris* and *P. aeruginosa* was assessed using field emission scanning electron microscopy [34]. Precisely, GO-AgNP-treated and -untreated catheter segments were placed in 12 well plates seeded with overnight grown cultures of *C. auris* and *P. aeruginosa* for 48 h at 37 °C. Post-incubation, the catheters were washed thrice with PBS to remove unbound cells, followed by fixation with 4% formaldehyde at room temperature for 1 h. The biofilms were then dehydrated using a gradient of ethanol in the following order: 30%, 50%, 70%, 90%, and 100% (30 min each). The catheter segments were air-dried, gold-coated, and viewed under the SU 8010 (Hitachi, Tokyo, Japan) ultra-high-resolution FE-SEM at the Sophisticated Analytical Instrumentation Facility (SAIF), Panjab University, Chandigarh, India.

### 2.6. Hemocompatibility of GO-AgNP-Functionalized Catheters

The nanocomposite-impregnated catheters were investigated for hemocompatibility using sheep erythrocytes in vitro [35]. Using a modified protocol, a 2% sheep red blood cell (RBC) suspension was prepared in normal saline, followed by the addition of a catheter segment (GO-AgNP-impregnated and un-impregnated) to 1 mL of RBC suspension in a sterile vial, which was incubated at 37 °C for 3 h under static conditions. Post-incubation, the catheter segment was removed and the vial was centrifuged at 2000× *g* for 5 min at 4 °C. The resulting supernatant was transferred to a fresh vial and the absorbance values were recorded at 545 nm. The concentration of hemoglobin released (in mg/mL) was determined following the normalization of A_545_ values to a hemoglobin standard curve. An RBC suspension containing 20% DMSO was used as positive hemolytic control, while those without DMSO served as negative control for the experiment. The hemolytic percentage/index was calculated as follows: hemolysis (%) = (concentration of hemoglobin released in supernatant/total hemoglobin concentration in tube) × 100. Based on the criteria proposed by The Standard Practice for Assessment of Hemolytic Properties of Materials (ASTM F 756) reports (2008), the nanocomposite formulation was classified as non-hemolytic (H index ≤ 2.0), mildly hemolytic (2.0 ≤ H index ≥ 5.0), and highly hemolytic (H index ≥ 5.0).

### 2.7. Biocompatibility of Nanocomposite-Impregnated Catheters

A modified MTT (3-[4,5-dimethylthiazol-2-yl]-2,5-diphenyl tetrazolium bromide)-based assay was used to assess the cytotoxicity of GO-AgNPs [36]. Human embryonic kidney cells (HEK 293) were revived in DMEM supplemented with 10% fetal bovine serum, 100 U/mL of ampicillin, and 100 mg/mL streptomycin in a humidified incubator, with 5% CO_2_ at 37 °C, as described previously [37]. From a T25 flask showing 80–90% confluency, HEK cells were trypsinized and subjected to staining with 0.01% trypan blue to determine the viable cell count. The cell suspension was then diluted in DMEM and HEK cells were seeded in a 96-well plate at a density of 1 × 10^6^ cells/well and incubated at 37 °C for 24 h. Post-incubation, the spent medium was aspirated and replaced with 100 μL of fresh 10% DMEM. HEK cells were then subjected to treatment with various catheters, including un-impregnated (control) and GO-AgNP-impregnated (test) catheters, for 4 h at 37 °C. Untreated HEK cells served as the positive growth control for the experiment. Post-incubation, the biomaterial was removed using sterile forceps and the spent media was replaced with fresh 10% DMEM. Subsequently, 10 μL of MTT labelling reagent (Roche, Mannheim, Germany) was added to each well and the plate was incubated at 37 °C for 3 h, followed by the addition of 100 μL of a solubilization buffer to solubilize the formazan crystals and incubation for 2 h at 37 °C. The absorption values of the samples were measured at 570 nm using a microplate reader (Bio-Rad, Hercules, CA, USA). Cell viability (%) was then estimated as the ratio of mean values of A_570_ values of the test samples to that of untreated HEK cells (positive growth control).

## 3. Results and Discussion

### 3.1. Cell Surface Hydrophobicity 

**Figure 1:**
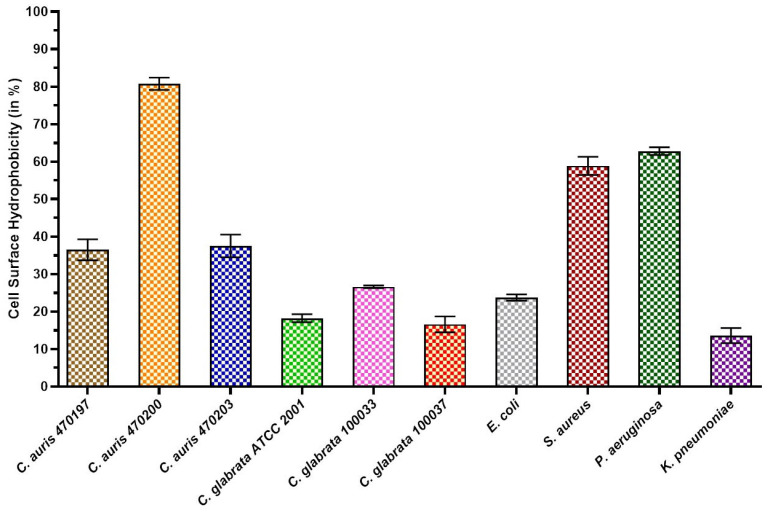
Cell surface hydrophobicity of test microorganisms used in this study.

All the microbial strains exhibited a hydrophobic nature, as we analyzed cell surface hydrophobicity using hydrocarbon xylene. *C. auris* NCCPF 470200 exhibited the highest percentage of hydrophobicity on its cell surface (80.7 ± 0.4%) among the three strains of *C. auris*, whereas NCCPF 470197 and 4710203 displayed moderate hydrophobicity (36.4 ± 0.5% and 37.4 ± 0.3%, respectively) (Figure 1). The hydrophobicity percentages of *C. glabrata* NCCPF 100033, 100037, and ATCC 2001 were found to be low (18.2 ± 0.02%, 26.5 ± 0.3%, and 16.5 ± 0.3%, respectively). Moreover, notable variations in percentage hydrophobicity were observed across different test strains (intraspecies) of *C. auris*, while the same was insignificant for *C. glabrata*. Interestingly, notable differences in cell surface hydrophobicity patterns were detected between *C. auris* and *C. glabrata* [38]. *S. aureus* (58.8 ± 0.2%) and *P. aeruginosa* (62.8 ± 0.2%) showed the greatest affinity for xylene among the bacterial strains. The lowest percentages of cell surface hydrophobicity (23.7 ± 0.2% and 13.5 ± 0.3%, respectively) were found in *E. coli* and *K. pneumoniae*. All data are displayed as means and standard deviations, with *p*-values ≤ 0.05.

### 3.2. Characterization of Biosynthesized Go-AgNPs

#### 3.2.1. UV–Visible Spectroscopy

**Figure 2:**
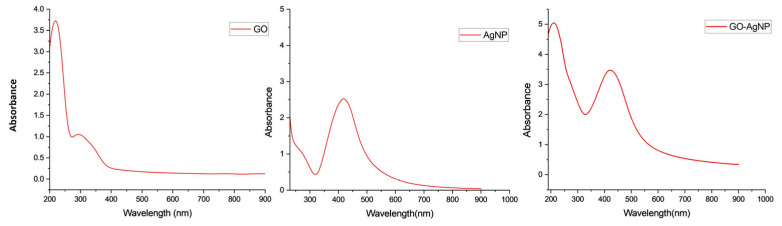
The GO-AgNP spectrum shows the characteristics peak of both GO and AgNP.

The UV–vis. spectra of pristine GO exhibited a maximum peak at 220 nm and a shoulder at 298 nm. The peak with the highest intensity, around 230 nm, corresponds to the π-π* transitions of aromatic C-C bonds, whereas a shoulder around 300 nm corresponds to the *n*-π* transitions of C=O bonds. For GO, the transition from π-π* is associated with the delocalized bonding network in the graphene plane. On the other hand, the transition from *n*-π* is linked to the oxygen-containing functional groups present on the graphene surface [39]. The successful reduction of AgNO_3_ to AgNPs on the GO matrix was confirmed by the appearance of the maximum absorption band at 420 nm, corresponding to the characteristic surface plasmon band of AgNPs at room temperature [40]. The peak maxima of GO shifted from 220 nm to 210 nm, while the shoulder, at 298 nm, disappeared (Figure 2). The observations for GO-AgNP were consistent with the observations made for pristine GO and AgNP.

#### 3.2.2. Fourier Transform Infrared Spectroscopy (FTIR) 

**Figure 3:**
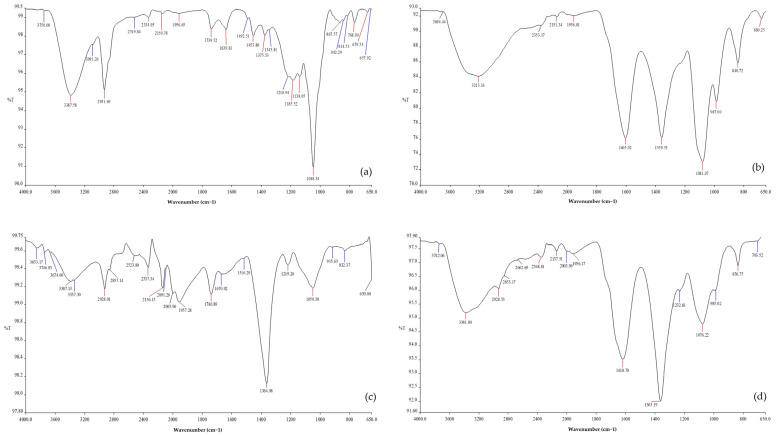
FTIR spectra of (**a**) *T. govanianum* plant extract, (**b**) GO, (**c**) AgNP, and (**d**) GO-AgNP.

The capping of the extract on the AgNPs and GO-AgNP surfaces was verified using FTIR spectroscopy, which also helped in identifying the functional groups of biomolecules involved in Ag^+^ bio-reduction, along with GO-AgNP capping and stabilization. The analysis was conducted at CIL (Central Instrumentation Laboratory), Panjab University, Chandigarh, using a Perkin Elmer-Spectrum RX-IFTIR in ATR mode at a resolution of 1 cm^−1^ and a scan range of 4000–650 cm^−1^. There were noticeable similarities between the transmittance peaks in the FTIR spectrum obtained for GO, AgNP, and GO-AgNP (Figure 3). The FTIR spectrum of GO and GO-AgNP displayed the presence of a broad and strong peak at wavenumbers 3213 cm^−1^ and 3381 cm^−1^ (OH-stretching vibration mode), respectively [41]. This broad peak at ~3500 cm^−1^ corresponds to the presence of hydroxyl groups and water molecules [17]. A carbonyl group C=O bond was present, as evidenced by intense peaks at wavenumbers 1605 cm^−1^ and 1359 cm^−1^ for GO, and 1618 cm^−1^ and 1360 cm^−1^ for GO-AgNP [17]. The peak at approximately 1600 cm^−1^ corresponds to the C=C from the sp^2^ hybrid domain and the C=O stretching of the carboxylic group on the basal plane, indicating GO’s overall negative charge [41]. In addition, there were small transmittance peaks in the region of wavenumbers 1956.81 cm^−1^ and 1956.17 cm^−1^, suggesting the presence of a COO bond (carboxyl group). The stretching vibrations of C-OH and C-O were represented by the peaks at ~1300 cm^−1^ and ~1100 cm^−1^, respectively [41,42]. The presence of peaks at 985 cm^−1^ and 987 cm^−1^ also corresponds to the presence of the C-O bond in GO and GO-AgNP, respectively. Small bands, at ~1200 cm^−1^, indicate the presence of the C-O-C bond (epoxy group) [17]. Moreover, peaks obtained at 2348, 1618, 1363, 1076, and 836 cm^−1^ were in accordance with the spectra obtained for the *T. govanianum* extract, suggesting the presence of capping agents (phytomolecules) present within the nanoparticles. In addition, the absorption bands at 3381.80 and 1363.19 cm^−1^ verified the presence and coating of silver nanoparticles on the GO surface (Figure 3d).

#### 3.2.3. EDS Analysis of Nanocomposite

**Figure 4:**
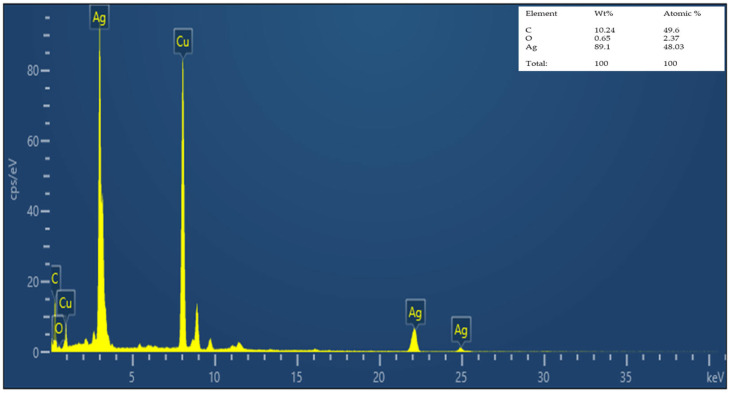
EDS spectrum of GO-AgNP.

The analysis of GO-AgNP using a TEM-equipped energy dispersive X-ray spectrometer (EDS) revealed that silver was successfully loaded onto the partially reduced graphene oxide (48.03% Ag), with no impurities found (Figure 4). The presence of less oxygen in GO-AgNP, as compared to pristine GO, indicates that GO has been successfully reduced to partially reduced GO. Similar findings have been recently reported in studies employing chemical-based methods [43] and green synthesis [23] of GBNs.

#### 3.2.4. Transmission Electron Microscopy (TEM) of GO-AgNPs

**Figure 5:**
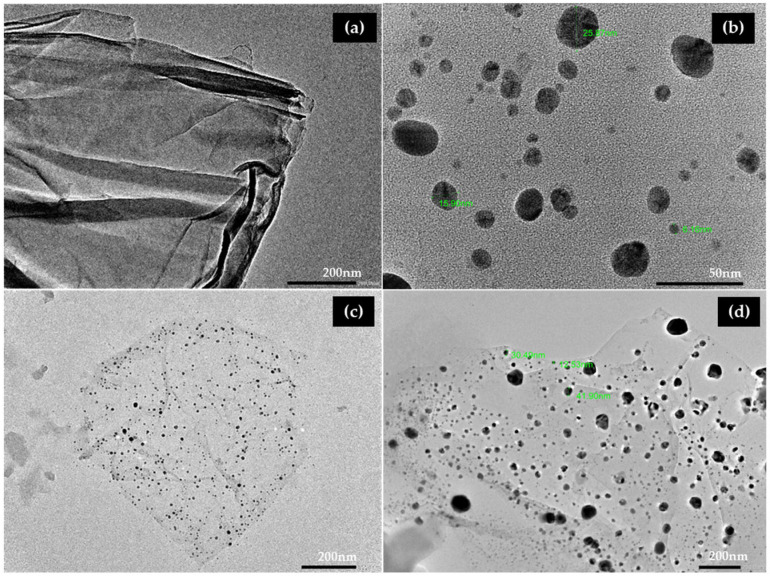
TEM micrographs of (**a**) pristine GO, (**b**) AgNP, and (**c**,**d**) GO-AgNP.

**Figure 6:**
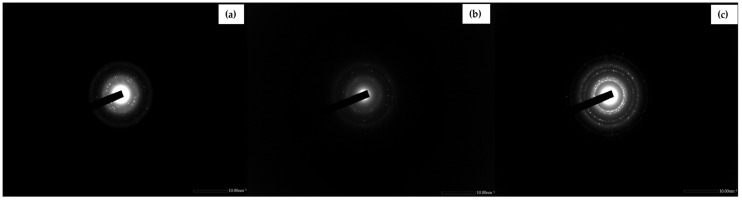
SAED images of (**a**) GO, (**b**) AgNP, and (**c**) GO-AgNP show the crystalline nature of the green-synthesized nanocomposite.

The morphological characteristics of pristine GO, AgNP, and GO-AgNP were observed using TEM (Figure 5). Graphene oxide suspension and GONP (GO + AgNO_3_ solution) were used as controls. The TEM images depicted that the synthesized graphene oxide nanosheets were very thin and transparent. Additionally, it demonstrated that the AgNPs were evenly dispersed across the surface of GO nanosheets, spherical, and small (10–100 nm) in size. The homogeneous distribution of silver nanoparticles, without any aggregation, demonstrated the successful synthesis of GO-Ag nanocomposites. The results were in accordance with findings reported by Li et al. [20]. SAED images of AgNP and GO-AgNP revealed a ring pattern, indicating that the material was polycrystalline, whereas GO demonstrated a spot pattern, indicating a single crystalline nature (Figure 6) [43].

### 3.3. In Vitro Antimicrobial Efficacy of GO-Ag Nanocomposites 

**Figure 7:**
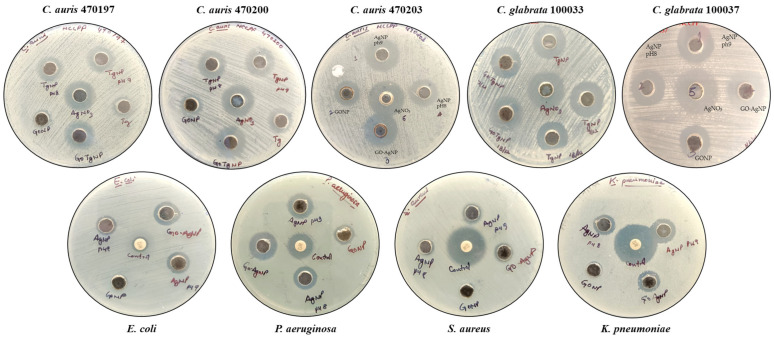
Antimicrobial potential of green-synthesized GO-AgNPs against test pathogens.

The antimicrobial activity of GO-AgNP was investigated against *C. glabrata* and *C. auris*, *E. coli*, *S. aureus*, *K. pneumoniae*, and *P. aeruginosa*. An agar-well diffusion assay was used to screen the antimicrobial potential of GO-AgNPs (Table 1). The pristine GO suspension did not show any antimicrobial activity on agar plates [20]. The biosynthesized GO-AgNPs exerted significant antimicrobial activity against *C. glabrata* and *C. auris*, as compared to bacteria (Figure 7). 

#### Determination of Minimum Inhibitory Concentration (MIC) and Minimum Lethal Concentration (MLC)

The biosynthesized GO-AgNPs showed antimicrobial activity when compared to AgNPs after 24 h of incubation against all the microorganisms. The antimicrobial activity varied at different concentrations of AgNPs and GO-AgNPs. The MIC values for GO-AgNPs (62.5–250 µg/mL) were found to be half of the values obtained in the presence of AgNP (31.25–125 µg/mL). A similar trend was observed in the case of MLC values ranging from 125 to 500 µg/mL and from 62.5 to 250 µg/mL, respectively (Table 2). The *C. glabrata* ATCC 2001 strain demonstrated the lowest MIC values for AgNP and GO-AgNP, which is in sync with the findings previously reported in the literature [23].

Silver nanoparticles (AgNPs) are potent antimicrobial agents, with the potential to serve as surface coatings to enhance the properties of biomaterials [44]. Silver nanoparticles, when used in combination with graphene oxide sheets, showed better antimicrobial and antibiofilm activity compared to AgNP [24]. The two-dimensional structure of the graphene and graphene derivatives contains corners and sharp edges, which are critical to their physicochemical interactions with microorganisms [45]. GO sheets can cause physical disruption by rupturing the cell wall, and inducing ROS-dependent oxidation stress [41,46]. As a result, the transport of silver ions across the microbial cell is enhanced [43]. Generally, AgNPs tend to undergo self-aggregation, which results in decreased antimicrobial potential and stability over time. To address this limitation, incorporating AgNPs onto the graphene matrix prevents the self-aggregation of Ag^+^ ions, consequently enabling their regulated release, enhanced stability, and prolonged antimicrobial activity [20].

### 3.4. Biofilm Inhibition

**Figure 8:**
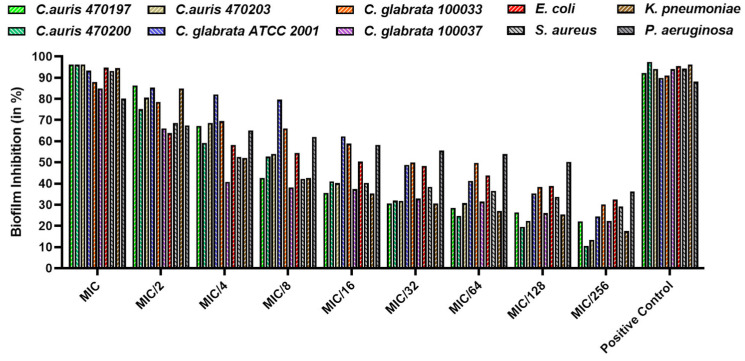
Inhibition of biofilm formation by GO-AgNP at MIC and sub-MIC concentrations.

The effect of GO-AgNPs was assessed on biofilm formation for all the microbial strains at both MIC and sub-MIC values. Interestingly, the microbial biofilms were inhibited by nearly 96% for *C. auris* and 84–93% for *C. glabrata* strains. Contrarily, the bacterial bio-films of *E. coli*, *S. aureus*, *K. pneumoniae*, and *P. aeruginosa* were significantly impeded, by 94%, 93%, 94%, and 80%, respectively, in reference to their respective positive controls (Figure 8).

### 3.5. GO-AgNP-Functionalized Catheters Avert Biofilm Formation by C. auris and P. aeruginosa

**Figure 9:**
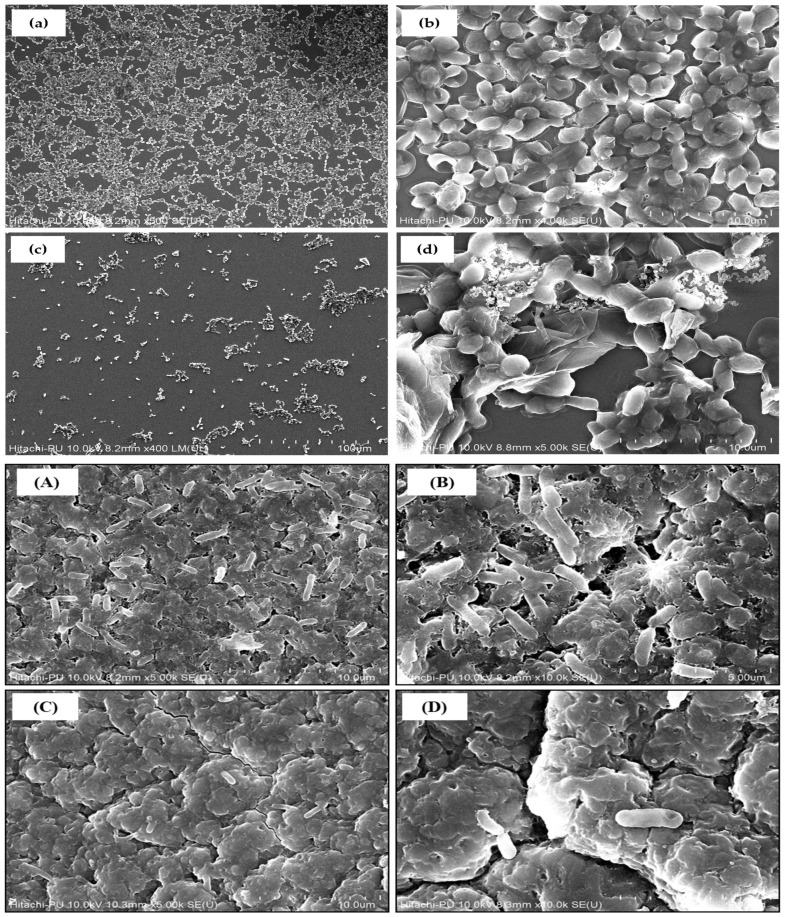
FE-SEM micrograph of biofilm inhibition in *C. auris* (**a**,**b**) control; (**c**,**d**) GO-AgNP-treated *C. auris* biofilm, (**a**–**d**) scale bar: 100 μm; (**A**,**B**) *P. aeruginosa* biofilm control; (**C**,**D**) GO-AgNP-treated biofilm. (**A**,**C**) scale bar: 10 μm; (**B**,**D**) scale bar: 5 μm.

Among the test pathogens, *C. auris* (fungal) and *P. aeruginosa* (bacterial) are known to form resilient biofilms that prevent penetration of antimicrobial drugs and effectively help in evading the host immune-system responses [20,29]. Based on the electron micrographs, *C. auris* and *P. aeruginosa* formed strong biofilms on untreated catheter segments, which were characterized by high cell densities and the presence of extrapolymeric substances around the cells (Figure 9). However, in the case of GO-AgNP-functionalized catheter segments, a notable reduction in adhered bacterial and yeast cells was observed, which was indicative of the biomaterial’s anti-fouling potential (Figure 9). In contrast to the treated groups, which displayed distorted appearances, rough cell surfaces, and intracellular component leakage as a result of plasmolysis, the biofilm in the control group retained its architectural integrity and had a clearly defined smooth cell surface [31,47]. Hence, our findings indicate that GO-AgNP-functionalized catheters effectively prevent cell adhesion and biofilm formation by *C. auris* and *P. aeruginosa*.

### 3.6. GO-AgNP-Functionalized Catheters Demonstrate Hemocompatibility and Biocompatibility In Vitro

**Figure 10:**
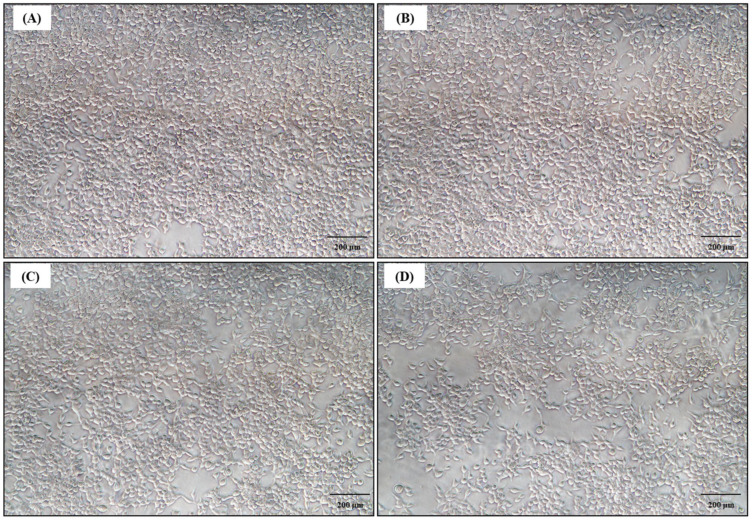
Microscopic view of HEK293 cells at 10× magnification: (**A**) untreated control, (**B**) untreated catheter, (**C**) GO-AgNP-functionalized catheter 1 mg/mL, (**D**) GO-AgNP-functionalized catheter 0.5 mg/mL.

Apart from possessing the desired pharmacological properties (antimicrobial), an ideal biomaterial must also exhibit a safe profile for its widescale application as a therapeutic agent [48]. Hence, we scrutinized the hemocompatibility and biocompatibility of GO-AgNP-functionalized urinary catheters using sheep erythrocytes and HEK293 cells in vitro, respectively. With respect to testing against erythrocytes, control catheters (un-impregnated) displayed hemocompatibility, with a hemolytic index of 0.705 ± 0.0623. Interestingly, catheters impregnated with the GO-AgNP nanocomposite at 1 mg/mL and 0.5 mg/mL showed moderate hemolysis, with hemolytic indices of 4.084 ± 0.151 and 2.659 ± 0.024, respectively. Since functionalized catheters were found to be hemocompatible in nature, we were intrigued to investigate their cytotoxic effects against HEK293 cells, as a function of MTT reduction, in reference to untreated cells. As anticipated, the control catheters exhibited a cell viability of 99.85 ± 2.057% (Figure 10A,B). On similar lines, GO-AgNP-functionalized catheters (1 mg/mL and 0.5 mg/mL) also demonstrated excellent biocompatibility towards HEK293 cells, with viability values of 92.45 ± 2.123% and 95.39 ± 5.465%, respectively, without altering the cell structure and morphology (Figure 10C,D). Several other investigations have also indicated similar findings in relation to the biocompatible nature of silver-doped graphene films [49] and graphene oxide–antimicrobial peptide nanoconjugates [50]. Hence, these findings strongly indicate that our GO-AgNP nanocomposite not only harbors antimicrobial prospects but also exhibits hemocompatibility and biocompatibility, thereby suggesting its safe profile for possible application in animal models.

## 4. Conclusions

With the aim of exploring alternate therapeutics to conventional antimicrobial drugs, this study was aimed at developing novel biomaterials comprising silver and graphene nanocomposites. Using *T. govanianum* extract, the green synthesis of graphene–silver nanocomposites was successfully undertaken and biophysically characterized. The biological activity of the nanocomposite against multidrug-resistant nosocomial bacteria, as well as against fungal pathogens, was also investigated, revealing promising antimicrobial prospects. Moreover, the nanocomposite also harbored remarkable anti-fouling properties against *C. auris*, *C. glabrata*, and *P. aeruginosa*, the major pathogens associated with robust biofilm-related infections. Further, the novel nanoformulation (GO-AgNP) functionalized-catheters displayed hemocompatibility and biocompatibility towards erythrocytes and HEK293 cells in vitro. Taken together, our findings indicate that the GO-Ag nanocomposite is an effective candidate against nosocomial pathogens and may explored for its possible applications in clinical settings after in vivo validation.

## Figures and Tables

**Table 1:** Antimicrobial potential in terms of zone of inhibition of biosynthesized nanocomposites against different microbial strains.

Test Microorganism	Zone of Inhibition (mm) Obtained with		
	GO-AgNPs	AgNO_3_	Distilled Water
*C. glabrata* ATCC 2001	16.0 ± 0.2	12.0 ± 0.2	ND
*C. glabrata* NCCPF 100033	18.0 ± 0.2	12.0 ± 0.2	ND
*C. glabrata* NCCPF 100037	17.0 ± 0.2	13.0 ± 0.2	ND
*C. auris* NCCPF 470197	17.5 ± 0.2	14.0 ± 0.2	ND
*C. auris* NCCPF 470200	18.0 ± 0.2	14.0 ± 0.2	ND
*C. auris* NCCPF 470203	17.0 ± 0.2	13.0 ± 0.2	ND
*E. coli*	10.0 ± 0.2	8.0 ± 0.2	ND
*S. aureus*	6.0 ± 0.2	6.0 ± 0.2	ND
*K. pneumoniae*	8.5 ± 0.2	5.0 ± 0.2	ND
*P. aeruginosa*	9.0 ± 0.2	5.0 ± 0.2	ND

ND = no zone of inhibition detected; diameter of the zone of inhibition (mm) (does not include the diameter of the well); for three replications, all data are displayed as means and standard deviations.

**Table 2:** MIC and MLC of biosynthesized silver nanoparticles and graphene–silver nanocomposites against different microorganisms using microbroth dilution method.

Microorganisms	MIC (µg/mL)		MLC (µg/mL)	
	AgNP	GO-AgNP	AgNP	GO-AgNP
*C. glabrata* ATCC 2001	62.5	31.25	125	250
*C. glabrata* NCCPF 100033	250	125	500	250
*C. glabrata* NCCPF 100037	125	62.5	250	125
*C. auris* NCCPF 470197	125	62.5	250	125
*C. auris* NCCPF 470200	125	62.5	250	125
*C. auris* NCCPF 470203	125	62.5	250	125
*E. coli*	125	62.5	250	125
*S. aureus*	125	62.5	250	125
*K. pneumoniae*	250	125	500	250
*P. aeruginosa*	125	62.5	250	125

## Data Availability

The authors have included all the primary data in the manuscript. Raw data will be made available on genuine/official request to the corresponding author.
